# *One Health* turns 10: A decade at the intersection of human, animal, and environmental health

**DOI:** 10.1016/j.onehlt.2025.101218

**Published:** 2025-09-26

**Authors:** Malcolm K. Jones, Dearbháile Morris, Huaiping Zhu, Aileen M. Marty

**Affiliations:** a School of Veterinary Science, The University of Queensland, Gatton 4343, and Division of Infection and Immunity, QIMR Berghofer Medical Research Institute, Herston 4006, Australia; b Antimicrobial Resistance and Microbial Ecology Group, School of Medicine, and Centre for One Health, Ryan Institute, University of Galway, Ireland; c Department of Mathematics and Statistics, Laboratory of Mathematical Parallel Systems (LAMPS) and Centre for Diseases Modelling (CDM), York University, Toronto, Ontario, M3J 1P3, Canada; d College of Arts, Sciences, and Education, Honors College, and Herbert Wertheim College of Medicine, Florida International University, Miami, Florida, USA

## 1 Why *One Health*, and why now?

The first issue of *One Health* was published in December 2015, marking a pivotal milestone for the integration of human, animal, and environmental health [1]. While earlier journals explored related disciplines, *One Health* was the first scholarly journal wholly dedicated to this integrative field.

In August 2025, as the journal compiles its 21st issue and surpasses 1000 published articles, its *Web of Science* Journal Impact Factor stands at 4.5, and its Scopus CiteScore is 7.7. Moreover, its mission—to unite researchers, practitioners, and policymakers from diverse backgrounds—has never been more crucial. The ongoing interest of researchers in the One Health domain underscores the long-held contention that human well-being cannot be considered in isolation from the environments we share, willingly or unwillingly, from other species, large and small.

## 2 Expanding the environmental dimension and emphasizing sustainable practice

One Health recognizes that human and animal health are inextricably linked to the health of the environment and to each other. Environmental degradation—through deforestation, pollution, biodiversity loss, and climate change—not only disrupts ecosystems but also increases the risk of infectious diseases and makes control far more difficult [2]. Sustainable practices such as ecosystem restoration, pollution reduction, and environmentally responsible regenerative agriculture are central to preventing future outbreaks and ensuring both food security and public health [3]. The increasing pressures to enhance animal production for human consumption raise the fraught issues of antimicrobial resistance that will plague the upcoming generations [4].

The journal has championed research into climate impacts on disease patterns [5], linking the spread of vector-borne and zoonotic diseases to environmental shifts. Studies have demonstrated how restoration and environmental stewardship can buffer disease emergence, highlighting environmental health as a lever for planetary resilience [6].

## 3 Food safety, security, and agricultural impacts

*One Health* foregrounds the impact of plant and animal diseases on food systems, food safety, and nutrition, particularly in low- and middle-income countries (LMICs) [7]. Notable recent reviews have addressed antimicrobial resistance (AMR) in animal production, foodborne zoonoses, and how best to implement integrated surveillance from farm to fork [8]. The intersection of sustainable agriculture and disease prevention is now a recurrent theme in our special issues and research agendas [9].

### 3.1 The ongoing and expanding issue of zoonotic infections

Human encroachment into wild spaces close to urban centers and their airports, coupled with ecotourism and climate-driven mobility of animals and humans, greatly raises the risks of zoonoses spreading rapidly on a global scale [27]. Some had predicted, as early as 2007, that SARS-CoV viruses were a time bomb [10] and the most likely cause of the ‘next’ pandemic would be due to a coronavirus. This foresight did not prepare us entirely for the emergence of SARS-CoV-2, its spread, socioeconomic impact, and anthropozoonotic (animal infections acquired from humans, also known as 'reverse zoonotic') transfers. The animal virus mPox (formerly monkeypox) remains a persistent threat to humans and may one day transform into a respiratory virus with unknown potential for disease [11]. The world is still in the middle of a massive epizootic of H5N1, and an H5N1 clade or other H5NX influenza A virus is poised to acquire the mutation in Segment 4 of the segmented viral RNA. This mutation would allow the viral hemagglutinin glycoprotein to bind with higher affinity to α2,6-linked sialic acid receptors abundant in human upper respiratory cells, altering a disease that is currently most serious in birds [12] to become a potentially devastating influenza pandemic.

## 4 Social, behavioral, and local community perspectives

Recognizing that successful One Health interventions depend as much on social context and community partnership as on laboratory breakthroughs, the journal increasingly showcases implementation science, behavioral research, and participatory approaches [13]. Community-based surveillance and indigenous/local knowledge are now acknowledged as crucial in devising sustainable health solutions that work in both LMICs and high-income countries (HICs) [14].

## 5 Landmark policy frameworks and the quadripartite organizations

The past decade has seen a new level of international collaboration. The ongoing work of the quadripartite organizations—World Health Organization (WHO), Food and Agriculture Organization of the United Nations (FAO), World Organisation for Animal Health (WOAH), and United Nations Environment Programme (UNEP)—has driven global action on threats like pandemics and AMR. The journal frequently publishes updates, consensus statements, and reviews of these collaborations, including the global quadripartite action plans and congresses that have shaped the field [15].

## 6 Breakthrough articles and special issues: showcasing impact

These topics, revealing the interrelated aspects of health, have been the ‘business’ of the *One Health* journal over the last 10 years. From the very first issue, which explored sea lions as sentinels of coastal organophosphate pollution, poultry diseases in rural communities, and MERS-CoV in camelids, *One Health* has published studies with real-world and global policy relevance. The annual number of papers published expanded rapidly with the onset of the COVID-19 pandemic. *One Health* took the important and correct decision to publish on COVID-19 as it is ‘a quintessential One Health issue’ [16].

Among many other publication achievements, the COVID-19 pandemic underscored our commitment to alert medical practitioners to the vast scope of animal research on coronaviral diseases [17,18]. One author countered the popular dialogues in media (read ‘hype’) in many countries of the Americas that were promulgating ivermectin as a panacea for COVID-19 [19]. The journal served as a rapid repository for research ranging from cross-species transmission (e.g., SARS-CoV-2) to evidence-based critiques of proposed therapeutics like ivermectin, which was ultimately not helpful, and remdesivir [20], which proved very helpful and became the first FDA-approved antiviral drug for the treatment of COVID-19 [21].

## 7 Policymakers and geopolitical concerns

During our time with the journal, it has been valuable to witness the growing international recognition of One Health as a guiding framework for both global and national policymaking (e.g., through the quadripartite collaborations of WHO, FAO, WOAH, and UNEP) [22]. Equally important is how national and local partnerships are translating these principles into practical One Health solutions to address a wide range of health threats.

The One Health arena is large and expanding, and challenges remain. After decades of positive advances against diseases of One Health concern, we anticipate that the domain will experience obstacles to these advances. An unanticipated challenge has been the massive decline in health-related funding from the United States of America (USA) and stagnation in funding from several other high-income nations for entrenched diseases, notably neglected tropical diseases (NTDs) that straddle human, animal, and environmental spheres [23]. The USA has enacted massive direct funding cuts; while in other high-income countries, overall investment has largely remained flat or just not kept pace with other health priorities, resulting in insufficient growth to meet the global needs for these diseases as summarized in recent policy reviews [23]. This pullback endangers global progress and underscores the need for new financing mechanisms and partnerships. Thus, whatever the reasons for declining support, we share the alarm of our colleagues in the NTD space and echo the call for novel ways to fund and combat entrenched diseases that affect humans and animals.

The One Health approach to problem-solving is a vital component in the fight against diseases. We need to develop top-down, multi-disciplinary approaches to challenges of LMICs and HICs alike. *One Health* serves as a critical platform for bringing together scientific evidence and expert voices across human, animal, and environmental health. By showcasing data-driven research, policy analyses, and success stories, the journal works to inform not only scientists and medical providers but also policymakers, funders, and stakeholders of the urgent need and proven impact of integrated approaches to tackle zoonotic, pandemic, and NTDs. This visibility can drive renewed commitment, evidence-based decision-making, and targeted funding needed to reverse recent declines in support for health research, and in work on NTDs, HIV, and other entrenched health threats

## 8 Advocating for interdisciplinary, equitable, and sustainable solutions

*One Health* continues to champion top-down and bottom-up, multidisciplinary strategies that bring together veterinarians, physicians, environmental and social scientists, plant health experts, modelers, data scientists, economists, sociologists, and especially local communities [[24], [25], [26]]. Equitable approaches that address the burden on the world's most vulnerable are essential in an era of climate instability, AMR, and declining philanthropic funding.

## 9 Conclusion

As *One Health* enters its second decade, the journal remains steadfast in supporting, connecting, and amplifying voices striving for a world where the health of people, animals, plants, and the environment is recognized as one and indivisible. We pledge to remain a forum for groundbreaking research, a trusted venue for policy dialogue, and an advocate for sustainable and just solutions to the world's most pressing health challenges. The editors and publishers of *One Health* look forward to supporting the global community as we address the persistent challenges that plague us in our environments. The Quadripartite's One Health Joint Plan of Action (2022-2026) establishes six action tracks that now serve as a foundation for implementation across countries. We see the journal's next decade advancing these tracks through evidence synthesis, policy analysis, and practice-focused case studies [22].

## CRediT authorship contribution statement

**Malcolm K. Jones:** Conceptualization, Writing – original draft, Writing – review & editing. **Dearbháile Morris:** Writing – review & editing. **Huaiping Zhu:** Writing – review & editing. **Aileen M. Marty:** Writing – original draft, Writing – review & editing.

## Funding

No funding was received for this editorial.

## Declaration of competing interest

*Given their role as Editors of One Health, Malcolm Jones, Dearbháile Morris, Huaiping Zhu and Aileen* *Marty* *had no involvement in the peer-review of this article and had no access to information regarding its peer-review. Full responsibility for the editorial process for this article was delegated to another journal editor.*

## Data Availability

No data was used for the research described in the article.
